# Association of Diabetes With Central Corneal Thickness Among a Multiethnic Asian Population

**DOI:** 10.1001/jamanetworkopen.2018.6647

**Published:** 2019-01-04

**Authors:** Xiao-Yang Luo, Wei Dai, Miao-Li Chee, Yijin Tao, Jacqueline Chua, Nicholas Y. Q. Tan, Yih-Chung Tham, Tin Aung, Tien Yin Wong, Ching-Yu Cheng

**Affiliations:** 1 Guangdong Eye Institute, Guangdong Academy of Medical Sciences, Department of Ophthalmology, Guangdong General Hospital, Guangzhou, China; 2 Singapore Eye Research Institute, Singapore National Eye Centre, Singapore; 3 Department of Ophthalmology, The First Affiliated Hospital of Kunming Medical University, Kunming, China; 4 Ophthalmology & Visual Sciences Academic Clinical Program, Duke–National University of Singapore Medical School, Singapore; 5 Department of Ophthalmology, Yong Loo Lin School of Medicine, National University of Singapore, Singapore

## Abstract

**Importance:**

Thicker or thinner central corneas may lead to either overestimation or underestimation of intraocular pressure, which is the most important causal and treatable risk factor for glaucoma. However, the findings on the associations between diabetes, random glucose, and glycated hemoglobin A_1c_ (HbA_1c_) with central corneal thickness (CCT) are conflicting.

**Objective:**

To evaluate the associations between diabetes, random glucose, and HbA_1c_ with CCT in a multiethnic Asian population.

**Design, Setting, and Participants:**

Cross-sectional analysis of the Singapore Epidemiology of Eye Diseases (SEED) Study conducted from 2004 to 2011. A total of 10 033 Chinese, Malay, and Indian individuals 40 years or older residing in Singapore were recruited. Participants with incomplete information on diabetes status (448 participants), prior refractive or cataract surgery (1940 eyes), and corneal edema or dystrophy (29 eyes) were excluded. A meta-analysis was conducted to estimate the overall association of diabetes with CCT.

**Exposures:**

Standardized clinical examinations and interviewer-administered questionnaire to collect information about demographic, systemic, and ocular factors.

**Main Outcomes and Measures:**

Measurement of CCT using ultrasound pachymetry.

**Results:**

A total of 8846 adults (mean [SD] age, 57.9 [9.9] years; 4447 women [50.3%]) (17 201 eyes) were included in the final analyses. The CCT profile was similar among participants with and without diabetes (mean [SD] CCT, 545.3 [33.7] μm vs 544.8 [33.9] μm; *P* = .39). Following adjustments of age, sex, ethnicity, corneal curvature, axial length, and body mass index, CCT was a mean (SD) of 4.9 (0.8) μm (95% CI, 3.3-6.5 μm) thicker in patients with diabetes than those without diabetes. Multivariable analyses also showed that thicker CCT was associated with higher random glucose (per 10 mg/dL [to convert to mmol/L, multiply by 0.0555], β = 0.3; 95% CI, 0.2-0.4) and higher HbA_1c_ (per percentage, β = 1.5; 95% CI, 1.0-2.1) (all *P* < .001). These associations were significant in the subgroup with diabetes but not in the subgroup without diabetes. A meta-analysis including 12 previous population- and clinical-based studies showed that CCT was 12.8 μm (95% CI, 8.2-17.5 μm) thicker in eyes of patients with diabetes.

**Conclusions and Relevance:**

These findings suggest that diabetes and hyperglycemia were associated with thicker CCT. This study provides useful information on the interpretation of intraocular pressure in patients with diabetes.

## Key Points

**Question:**

Are diabetes status, random glucose, and hemoglobin A_1c_ associated with central corneal thickness (CCT) measurement?

**Findings:**

In this cross-sectional study of 8846 multiethnic Asian adults, diabetes and glucose and hemoglobin A_1c_ levels were associated with thicker CCT; these associations were significant in the subgroup with diabetes but not in the subgroup without diabetes. A meta-analysis including 12 previous studies showed that CCT was 12.8 μm thicker in eyes of patients with diabetes.

**Meaning:**

Findings from this study are important for CCT determination and may provide useful information on the interpretation of intraocular pressure in patients with diabetes.

## Teaser

This cross-sectional study investigates the association of diabetes, random glucose levels, and hemoglobin A_1c_ concentration with central corneal thickness in a multiethnic population of Asian adults in Singapore.

## Introduction

Diabetes mellitus is a serious and increasingly prevalent health problem worldwide due to sedentary lifestyle and population aging. Several studies have found that diabetes is a risk factor for primary open-angle glaucoma.^1,2,3,4,5^ Limited data from a population-based study showed that individuals with diabetes had thicker corneas.^6^ In this regard, central corneal thickness (CCT) has also been demonstrated to be associated with the onset and progression of glaucoma.^7^ Moreover, thicker or thinner central corneas may lead to either overestimation or underestimation of intraocular pressure (IOP),^8,9^ which is the most important causal and treatable risk factor for glaucoma. Previous studies indicated that CCT profile affects Goldmann applanation tonometry–measured IOP.^10,11^ This is especially so for eyes with CCT greater than 550 μm; every 25 μm increase in CCT was associated with 1 mm Hg change in IOP.^12^ This further suggests the importance of taking CCT profile into account when interpreting IOP measurement. Taken together, accurate determination of CCT is important in the context of glaucoma diagnosis and management.^13,14^

In clinical practice, CCT is widely regarded as a static parameter and is often only measured once during a patient’s long-term follow-up. However, several studies suggest that diabetic status and serum glucose concentrations may affect CCT measurement, thus potentially affecting IOP measurement. Nevertheless, the reported associations between serum glucose concentrations and CCT have not been consistent. Some studies demonstrated that higher serum glucose was linked with thicker CCT,^6,15^ while others did not observe significant association between serum glucose and CCT.^16^ Furthermore, glycated hemoglobin A_1c_ (HbA_1c_), which provides information about average serum glucose level for the past 2 to 3 months, is widely accepted as an important indicator of long-term glycemic control. Some studies have reported that higher HbA_1c_ was associated with thicker CCT,^6,15^ while 1 study found no correlations between the 2.^17^

In view of the conflicting findings, further elucidation in this area is warranted. Moreover, very few studies specifically evaluated serum glucose and HbA_1c_ level and their associations with CCT. Hence, the purpose of this study was to evaluate the association of diabetes, random glucose, and HbA_1c_ with CCT in a population-based study of a multiethnic Asian cohort of Malay, Indian, and Chinese individuals. We further performed a meta-analysis to assess the overall association between diabetes and CCT. Findings from this study may provide useful information on more accurate CCT determination, which is essential for interpretation of IOP in patients with diabetes.

## Methods

### Study Population

The Singapore Epidemiology of Eye Diseases (SEED) Study is a population-based study comprising 3 major ethnic groups in Singapore: Malay (Singapore Malay Eye Study, 2004-2006), Indian (Singapore Indian Eye Study, 2007-2009), and Chinese (Singapore Chinese Eye Study, 2009-2011). This study was approved by the SingHealth Centralized institutional review board and adhered to the Declaration of Helsinki. Written informed consent was obtained from all participants. Detailed methodology for the SEED Study has been previously published.^18,19^ A total of 10 033 participants (response rate, 75.6%) aged 40 to 80 years were enrolled in the SEED Study, which comprised 3280 Malay participants (response rate, 78.7%), 3400 Indian participants (response rate, 75.6%), and 3353 Chinese participants (response rate, 72.8%). Ethnicity was classified according to the individual’s National Registration Identity Card. Exclusion criteria included incomplete information on diabetes status, prior refractive or cataract surgery, and corneal edema or dystrophy. Of the 10 033 participants, we first excluded 448 participants with incomplete information on diabetes status. We excluded eyes with prior refractive or cataract surgery (1940 eyes) and corneal edema or dystrophy (29 eyes). This study followed the Strengthening the Reporting of Observational Studies in Epidemiology (STROBE) reporting guideline for reporting cross-sectional studies.

### Ocular Examination

All participants underwent standardized ocular examinations at the Singapore Eye Research Institute.^18,19^ The CCT of each eye was measured by an ultrasound pachymeter (Advent; Mentor O & O Inc), and the median reading of 5 measurements was used for analysis. Goldmann applanation tonometry (Haag-Streit) was used to measure IOP before pupil dilation. Autorefraction, keratometry, and corneal curvature were measured by autorefractor (Canon RK-5 Auto Ref-Keratometer; Canon Inc Ltd). Axial length was measured by noncontact partial coherence interferometry (IOLMaster V3.01; Carl Zeiss Meditec AG). Glaucoma was defined according to the International Society for Geographical and Epidemiologic Ophthalmology criteria. Ocular hypertension was defined as IOP greater than 21 mm Hg.

### Other Measurements

An interviewer-administered questionnaire was used to collect demographic data, lifestyle risk factors (eg, smoking), education level, medical history (eg, history of diabetes or hypertension), ocular history (eg, cataract surgery and refractive surgery), and medication use from all participants.^18^ Each participant had height and weight measurements, which were used to determine the body mass index (BMI), calculated as weight in kilograms divided by height in meters squared. Blood pressure was measured using a digital automatic blood pressure monitor (Dinamap model Pro100V2; Criticon GmbH). Blood samples (40 mL) were collected for measurements of random glucose, HbA_1c_, and cholesterol concentrations. Participants were classified as having diabetes if they met any of the following criteria: random glucose levels 200 mg/dL or higher (to convert to mmol/L, multiply by 0.0555), a self-reported use of medication for diabetes, physician diagnosis of diabetes, or HbA_1c_ 6.5% or more of total hemoglobin.

### Statistical Analysis

Analyses were performed using Stata statistical software version 14.0 (StataCorp). Comparisons of characteristics between participants with and without diabetes were performed using χ^2^ tests and *t* tests for categorical and continuous data as appropriate. Associations between ocular and systemic factors with CCT were assessed using linear regressions models. Models were initially adjusted for age, sex, and ethnicity, and then further adjusted for corneal curvature, axial length, and BMI. The selection of covariate adjustment was based on clinical knowledge. Subgroup analyses stratified by diabetes status were performed to examine the consistency of the association of random glucose and HbA_1c_ with CCT. To account for the correlation between pairs of eyes for each individual, linear regression with generalized estimating equation models with exchangeable correlation structures and a Gaussian link was used in the regression models. The *P* value (2-sided) for significance was set at less than .05.

### Meta-analysis on the Overall Association of Diabetes With CCT

A search of the PubMed and Web of Science databases was conducted for articles published between 1980 and 2016 using the keywords “central corneal thickness /CCT” and “diabetes/glycosylated haemoglobin/serum glucose.” We searched for articles that reported the distribution of CCT in participants with and without diabetes. After carrying out a more exhaustive and complete reading, 12 publications in English were finally identified. In addition to 3 of our SEED studies, we included 1 population-based study from Africa^20^ and 10 clinical-based studies^17,21,22,23,24,25,26,27,28,29^ in the meta-analysis. Sample size, mean, and SD were then extracted from these published articles to estimate the overall association of diabetes with CCT. We used the DerSimonian and Laird random-effects model to summarize CCT differences across studies. Heterogeneity between studies was quantified using the *I*^2^ statistic, which describes the proportion of total variation in study estimates attributable to heterogeneity. Overall, we included 3558 participants with diabetes and 11 342 participants without diabetes in the meta-analysis. This study followed the Preferred Reporting Items for Systematic Reviews and Meta-analyses (PRISMA) reporting guideline to report the meta-analysis (eFigure 1 in the Supplement).

## Results

In total, 8846 participants (mean [SD] age, 57.9 [9.9] years; 4447 women [50.3%]) (17 201 eyes) were included in the analysis (2993 Malay [33.8%], 2923 Indian [33.0%], and 2930 Chinese [33.1%] individuals). Of these, 2599 (29.4%) had diabetes (960 Malay [36.9%], 1137 Indian [43.7%], and 502 Chinese [19.3%] participants) (eFigure 2 in the Supplement).

Participants’ demographic, systemic, and ocular characteristics are summarized in Table 1. Overall, participants with diabetes were more likely to be older, have Indian ethnicity, have fewer years of formal education, be past smokers, and have higher systolic blood pressure, BMI, random glucose level, and HbA_1c_ concentrations but lower serum cholesterol. In ocular characteristics, those with diabetes had higher IOP, steeper corneal curvature, and shorter axial length, and were more likely to have glaucoma and ocular hypertension but were less likely to have myopia. The CCT profile was similar among participants with vs without diabetes (mean [SD] CCT, 545.3 [33.7] μm vs 544.8 [33.9] μm; *P* = .39).

**Table 1.** Participants’ Demographic, Systemic, and Ocular Characteristics

Characteristics	Patients With Diabetes (n = 2599)	Patients Without Diabetes (n = 6247)	*P* Value^a^
**Participant Specific**			
Age, y, mean (SD)	60.6 (9.6)	56.8 (9.8)	<.001
Male, No. (%)	1319 (50.8)	3080 (49.3)	.22
Ethnicity, No. (%)			
Malay	960 (36.9)	2033 (32.5)	<.001
Indian	1137 (43.8)	1786 (28.6)	
Chinese	502 (19.3)	2428 (38.9)	
Education level, No. (%)			
≤Primary	1774 (68.5)	3452 (55.3)	<.001
≥Secondary	816 (31.5)	2789 (44.7)	
Smoking status, No. (%)			
Never	1785 (68.8)	4327 (69.3)	<.001
Current	364 (14.0)	1099 (17.6)	
Past	445 (17.2)	815 (13.1)	
Blood pressure, mm Hg, mean (SD)			
Systolic	145.2 (22.3)	136.9 (21.2)	<.001
Diastolic	78.7 (10.6)	78.6 (10.5)	.54
Body mass index, mean (SD)^b^	27.0 (4.8)	24.9 (4.5)	<.001
Cholesterol, mg/dL, mean (SD)			
Total	201.8 (48.6)	214.5 (40.5)	<.001
HDL	44.2 (12.7)	49.6 (14.6)	<.001
LDL	124.3 (39.4)	134.9 (35.5)	<.001
Random glucose, mg/dL, mean (SD)	178.1 (86.6)	99.1 (20.4)	<.001
HbA_1c_, % of total hemoglobin, mean (SD)	7.8 (1.7)	5.7 (0.4)	<.001
**Eye Specific**			
Eyes, No.	4971	12 230	
Central corneal thickness, μm, mean (SD)	545.3 (33.7)	544.8 (33.9)	.39
Spherical equivalent refraction, diopters, mean (SD)	−0.08 (2.24)	−0.39 (2.40)	<.001
Intraocular pressure, mm Hg, mean (SD)	16.0 (3.3)	14.9 (3.2)	<.001
Corneal curvature, mm, mean (SD)	7.62 (0.26)	7.65 (0.26)	<.001
Axial length, mm, mean (SD)	23.5 (1.1)	23.7 (1.2)	<.001
Glaucoma, No. (%)	126 (2.5)	222 (1.8)	.002
Ocular hypertension, No. (%)	145 (2.9)	184 (1.5)	<.001

Abbreviations: HbA_1c_, glycated hemoglobin A_1c_; HDL, high density lipoprotein; LDL, low density lipoprotein.

SI conversion factors: To convert total cholesterol, HDL cholesterol, and LDL cholesterol to mmol/L, multiply by 0.0259 and random glucose to mmol/L, multiply by 0.0555.

^a^ *P* value was based on χ^2^ or independent *t* test where appropriate.

^b^ Calculated as weight in kilograms divided by height in meters squared.

After adjusting for age, sex, and ethnicity, participants with diabetes had CCT that was a mean (SD) of 5.2 (0.8) μm thicker compared with participants without diabetes (*P* < .001) (Table 2). These associations were similar in respective ethnic groups (Table 2). Moreover, higher IOP was observed in participants with diabetes and was associated with thicker CCT across 3 ethnicities. A 3.5% variation in IOP was associated with CCT, 0.8% associated with HbA_1c_, and 0.6% associated with random glucose (data not shown in tables), indicating that CCT was associated with more IOP variation than HbA_1c_ and random glucose. When further adjusted for corneal curvature, axial length, and BMI, CCT was a mean (SD) of 4.9 (0.8) μm (95% CI, 3.3-6.5 μm) thicker in participants with diabetes than those without diabetes (*P* < .001). Multivariable analyses also showed that higher random glucose (per 10 mg/dL, β = 0.3; 95% CI, 0.2-0.4; *P* < .001) and higher HbA_1c_ levels (per %, β = 1.5; 95% CI, 1.0-2.1; *P* < .001) were associated with thicker CCT (Table 3). This association was similarly observed across the 3 ethnic groups. The multicollinearity diagnostics were examined based on the variance inflation factor of covariates included in multivariable models. The variance inflation factors for all covariates were less than 2, indicating that there were no high correlations between the covariates and, thus, no issue of multicollinearity within these models.

**Table 2.** Association of Systemic and Ocular Factors With Central Corneal Thickness, Stratified by Ethnicity^a^

Characteristics	β (95% CI)			
	Overall	Malay Ethnicity	Indian Ethnicity	Chinese Ethnicity
Eyes, No.	17 201	5854	5665	5682
Age (per decade)	−5.3 (−6.0 to −4.6)^b^	−5.6 (−6.6 to −4.5)^b^	−4.3 (−5.7 to −3.0)^b^	−5.9 (−7.2 to −4.6)^b^
Female	−0.3 (−1.6 to 1.1)	0.8 (−1.6 to 3.1)	−1.3 (−3.7 to 1.1)	−0.4 (−2.7 to 2.02)
Blood pressure (per 10 mm Hg)				
Systolic	0.3 (−0.1 to 0.6)	0.5 (0.0 to 1.1)^c^	0.2 (−0.3 to 0.9)	−0.1 (−0.8 to 0.5)
Diastolic	0.2 (−0.5 to 0.9)	0.4 (−0.7 to 1.4)	−0.1 (−1.3 to 1.1)	0.3 (−0.9 to 1.6)
Intraocular pressure, mm Hg	0.6 (0.5 to 0.8)^b^	0.4 (0.2 to 0.6)^b^	0.7 (0.5 to 1.0)^b^	1.0 (0.7 to 1.2)^b^
Corneal curvature, mm	5.7 (3.1 to 8.2)^b^	5.4 (1.0 to 9.8)^c^	9.6 (5.7 to 13.5)^b^	2.3 (−2.6 to 7.1)
Axial length, mm	1.2 (0.7 to 1.7)^b^	2.2 (1.1 to 3.3)^b^	1.3 (0.5 to 2.2)^c^	0.5 (−0.1 to 1.1)
Body mass index^d^	0.4 (0.2 to 0.6)^b^	0.5 (0.3 to 0.8)^b^	0.2 (−0.1 to 0.4)	0.6 (0.3 to 0.9)^b^
Diabetes	5.2 (3.7 to 6.8)^b^	4.9 (2.3 to 7.4)^b^	5.6 (3.1 to 8.1)^b^	4.9 (1.7 to 8.1)^c^
Random glucose (per 10 mg/dL)	0.3 (0.2 to 0.4)^b^	0.3 (0.1 to 0.4)^c^	0.3 (0.2 to 0.5)^b^	0.4 (0.1 to 0.7)^c^
Serum HbA_1c_ (per %)	1.7 (1.2 to 2.2)^b^	1.3 (0.6 to 2.1)^b^	1.8 (1.0 to 2.6)^b^	2.2 (0.8 to 3.7)^c^

Abbreviation: HbA_1c_, glycated hemoglobin A_1c_.

SI conversion factor: To convert random glucose to mmol/L, multiply by 0.0555.

^a^ Adjusted for age, sex, and, additionally, for ethnicity in the overall group.

^b^ *P* < .001.

^c^ *P* < .05.

^d^ Calculated as weight in kilograms divided by height in meters squared.

**Table 3.** Associations of Diabetes, Random Glucose, and HbA_1c_ With Central Corneal Thickness, Stratified by Ethnicity

Characteristic^a^	β (95% CI)^b^			
	Overall	Malay Ethnicity	Indian Ethnicity	Chinese Ethnicity
Eyes, No.	17 201	5854	5665	5682
Diabetes	4.9 (3.3-6.5)^c^	4.7 (1.9-7.5)^d^	5.6 (3.1-8.1)^c^	3.7 (0.3-7.0)^d^
Random glucose (per 10 mg/dL)	0.3 (0.2-0.4)^c^	0.2 (0.1-0.4)^d^	0.3 (0.2-0.5)^c^	0.4 (0.1-0.6)^d^
Serum HbA_1c_ (per %)	1.5 (1.0-2.1)^c^	1.2 (0.4-2.0)^d^	1.8 (1.0-2.6)^c^	1.6 (0.1-3.1)^d^

Abbreviation: HbA_1c_, glycated hemoglobin A_1c_.

SI conversion factor: To convert random glucose to mmol/L, multiply by 0.0555.

^a^ Diabetes, random glucose, and HbA_1c_ were assessed separately, in respective multivariable model adjusted for age, sex, corneal curvature, axial length, body mass index, and ethnicity (only for overall group).

^b^ β for diabetes represents adjusted difference in central corneal thickness between persons with and without diabetes (reference group); β for random glucose and HbA_1c_ models represent adjusted change in central corneal thickness per unit change in glucose and HbA_1c_, respectively.

^c^ *P* < .001.

^d^ *P* < .05.

To examine whether the association of random glucose and HbA_1c_ with CCT differs among participants with and without diabetes, we further performed subgroup analyses stratifying by diabetes status. The associations of higher random glucose and HbA_1c_ with thicker CCT were observed in the subgroup with diabetes but not in the subgroup without diabetes (Table 4). Further analyses also demonstrated that participants with poorly controlled diabetes (HbA_1c_ ≥6.5%) had thicker CCT (β = 0.4; 95% CI, 0.1-0.7) compared with those with well-controlled diabetes (HbA_1c_ <6.5%) (data not shown). We further adjusted for duration of diabetes in a multivariable model among patients with diabetes (model 3 in Table 4). Higher random glucose (β = 0.2; 95% CI, 0-0.4; *P* = .01) and HbA_1c_ (β = 0.8; 95% CI, 0-1.6; *P* = .04) were still significantly associated with thicker CCT. Moreover, when further adjusted for the duration of diabetes in overall participants (duration of diabetes for participants without diabetes was defined as 0) in the multivariable model, diabetes (β = 3.4; 95% CI, 1.4-5.3), higher random glucose (β = 0.2; 95% CI, 0.1-0.4), and higher HbA_1c_ (β = 1.1; 95% CI, 0.5-1.7) were still significantly associated with thicker CCT (eTable in the Supplement). Taken together, this indicated that the observed associations were independent of diabetes duration.

**Table 4.** Association of Serum Glucose and HbA_1c_ With Central Corneal Thickness, Stratified by Diabetes Status

Characteristic^a^	β (95% CI)^b^				
	With Diabetes (n = 4971)			Without Diabetes (n = 12 230)	
	Model 1	Model 2	Model 3	Model 1	Model 2
Random glucose (per 10 mg/dL)	0.2 (0.0 to 0.3)^c^	0.2 (0.0 to 0.4)^c^	0.2 (0.0 to 0.4)^c^	0.1 (−0.3 to 0.5)	−0.1 (−0.5 to 0.4)
Serum HbA_1c_ (per %)	0.9 (0.1 to 1.6)^c^	0.8 (0.0 to 1.6)^c^	0.8 (0.0 to 1.6)^c^	1.9 (−0.5 to 4.3)	1.0 (−1.5 to 3.4)

Abbreviation: HbA_1c_, glycosylated hemoglobin A_1c_.

SI conversion factor: To convert random glucose to mmol/L, multiply by 0.0555.

^a^ Random glucose and HbA_1c_ were assessed separately, in respective multivariable model adjusted for age, sex, and ethnicity in model 1, and additionally, adjusted for corneal curvature, axial length, body mass index, and diabetes medication (among subgroup with diabetes) in model 2. Model 3 in subgroup with diabetes adjusts for variables in model 2 and, additionally, diabetes duration.

^b^ β represents adjusted change in CCT per unit change in random glucose and HbA_1c_, respectively.

^c^ *P* < .05.

In the meta-analysis, we included 14 eligible studies to examine the association between diabetes and CCT (Figure). In our analysis of 4 population-based studies and 10 clinical-based studies, participants with diabetes had 12.8 μm thicker CCT (95% CI, 8.2-17.5 μm; *P* < .001) than those without diabetes. There was no significant association between diabetes and CCT (β = 3.3; 95% CI, −0.4 to 7.0; *P* = .08) in population-based studies (3 Asian studies and 1 African study). Ten clinical-based studies^17,21,22,23,24,25,26,27,28,29^ showed that participants with diabetes had 19.3 μm thicker CCT (95% CI, 14.8-23.7 μm; *P* < .001) than those without diabetes.

**Figure.**
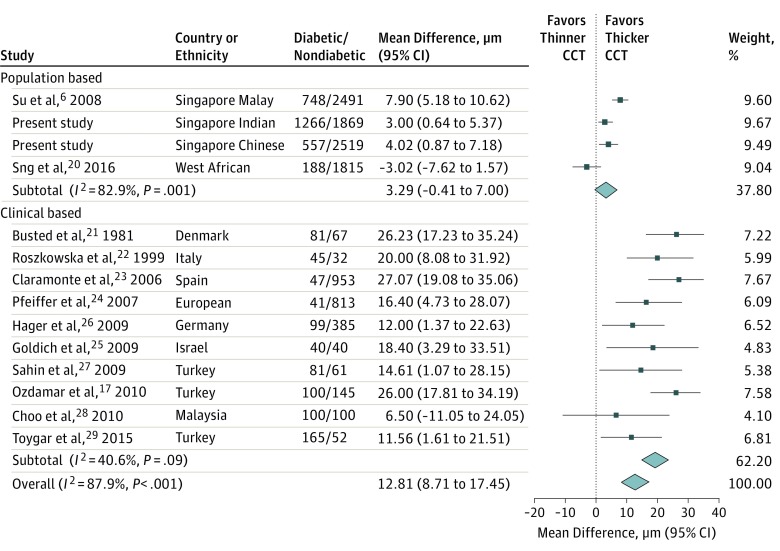
Forest Plot of Diabetes Impact on Central Corneal Thickness (CCT) Error bars represent the 95% confidence interval of mean difference in CCT between participants with and without diabetes. The weight contribution of each study to the pooled effect size is represented by the different sizes of the shaded box. Weights are from random-effects analysis.

## Discussion

In this large sample of a multiethnic population, we observed that diabetes, higher random glucose, and higher HbA_1c_ levels were associated with thicker CCT independent of potential confounders. In addition, these associations were only observed in the subgroup with diabetes and not in the subgroup without diabetes. Findings from this may provide useful information on the interpretation of IOP in clinical practice, especially in patients with diabetes.

In our study, we demonstrated that diabetes and hyperglycemia were associated with thicker CCT. Similar patterns were observed across Malay, Indian, and Chinese individuals in the study population. We have previously reported that diabetes, higher serum glucose, and higher HbA_1c_ were independently associated with thicker central corneas in Singapore’s Malay population.^6^ We also showed that participants with diabetes from a multiethnic Asian population had thicker corneas.^30^ Consistent with our findings, another population-based study^15^ also reported that diabetes was associated with thicker central corneas. However, some population-based studies^16,20,31^ failed to find a significant association between diabetes and CCT. The discrepancy may be partly due to different definitions of diabetes. For example, diabetes was defined by self-reported history in 2 studies,^20,31^ while fasting glucose level and oral glucose tolerance test were used to define diabetes status in other studies.^15,16^ The differences in the definition of diabetes may lead to misclassification, which could weaken the true association between diabetes and CCT.

Although the basis of the association of diabetes and hyperglycemia with CCT remains unknown, we postulate that excess glucose in the corneas of patients with diabetes leads to intracellular accumulation of sorbitol, which acts as an osmotic agent and results in the swelling of endothelial cells.^6^ In addition, endothelial pump function is affected by decreased adenosine triphosphate production from the Krebs cycle in the cornea of patients with diabetes.^28^ These speculated mechanisms eventually lead to morphological changes and swelling in corneas with diabetes. However, a previous study^28^ showed that although type 2 diabetes resulted in significant reduction of the corneal endothelial cell density, CCT was unaffected by diabetic status. Further investigation of the association between hyperglycemia and CCT will be beneficial to manage patients with these conditions. Thus, future studies to elucidate the underlying mechanisms are warranted.

In our meta-analysis, CCT was associated with diabetes in 3 Asian population-based studies but not in other studies.^20^ There are 3 possible reasons for this. First, different definitions of diabetes were used among these studies. In the African population-based study, diabetes status was self-reported by the participants, whereas in our study, we used random glucose level, HbA_1c_ level, medical history, and medication for diabetes to define diabetes. Self-reporting may underestimate the prevalence of diabetes, in which higher rates of undiagnosed diabetes have been reported in developing countries compared with developed countries.^32^ Second, there may be population heterogeneity between Asian and African individuals. Third, the sample sizes of the clinical-based studies were relatively small and, thus, may be subject to sampling bias.^33^ Sensitivity analysis for meta-analysis showed that after excluding the West African population, the heterogeneity among the remaining 3 Asian population-based studies was still high (*I*^2^ = 73.1%; *P* = .02). However, the pooled estimate among the 3 population-based studies became significant (β = 4.96; *P* = .001).

The strengths of our study include a large sample population of 3 ethnic groups with a high response rate (75.6%), as well as comprehensive and standardized assessment of systemic and ocular factors. This enabled us to adjust for relevant potential confounders comprehensively, which helped to substantiate the validity of our results.

### Limitations

Our study also has a few limitations. First, as this is a cross-sectional analysis, findings from our study cannot ascertain the actual causal relationship between diabetes, random glucose, HbA_1c_, and CCT. Second, we did not perform confocal microscopy of corneal endothelium, which would have provided potential insights on underlying corneal thickening changes among individuals with diabetes. Third, we only measured random glucose in this study, but did not measure fasting glucose. As random glucose is more variable compared with fasting glucose, caution should be taken when interpreting our results with previous studies that used different serum glucose measurement methods.

## Conclusions

In this large sample of a multiethnic Asian population, we found that diabetes and hyperglycemia were associated with greater CCT. Findings from this study are important for CCT determination and may provide useful information for the interpretation of IOP in patients with diabetes.

## Notes

Supplement. **eTable.** Associations of Diabetes, Random Glucose and HbA1c With Central Corneal Thickness, Stratified by Ethnicity **eFigure 1.** Flowchart of Information Through Different Phases of the Meta-analysis **eFigure 2.** Flowchart of Study Subjects
