# Supplementary material for: Association of Diabetes With Central Corneal Thickness Among a Multiethnic Asian Population
Source: JAMA Netw Open. 2019 Jan 4;2(1):e186647. doi: 10.1001/jamanetworkopen.2018.6647 (PMC6324536; doi:10.1001/jamanetworkopen.2018.6647)
Supplement: Supplement. — eTable. Associations of Diabetes, Random Glucose and HbA1c With Central Corneal Thickness, Stratified by Ethnicity eFigure 1. Flowchart of Information Through Different Phases of the Meta-analysis eFigure 2. Flowchart of Study Subjects [file jamanetwopen-2-e186647-s001.pdf]

Supplementary Online Content

Luo X-Y, Dai W, Chee M-L, et al. Association of diabetes with central corneal thickness among a multiethnic Asian population. *JAMA Netw Open*. 2019;2(1):e186647. doi:10.1001/jamanetworkopen.2018.6647

eTable. Associations of Diabetes, Random Glucose and HbA1c With Central Corneal Thickness, Stratified by Ethnicity

eFigure 1. Flowchart of Information Through Different Phases of the Meta-analysis

eFigure 2. Flowchart of Study Subjects

This supplementary material has been provided by the authors to give readers additional information about their work.

© 2019 Luo X-Y et al. *JAMA Network Open.*

eTable. Associations of diabetes, random glucose and HbA1c with central corneal thickness, stratified by ethnicity.

**Characteristics†**	**Overall (n = 17,201)** ***β*** **(95% CI)‡**	**Malays (n = 5,854)** ***β*** **(95% CI)‡**	**Indians (n = 5,665)** ***β*** **(95% CI)‡**	**Chinese (n = 5,682)** ***β*** **(95% CI)‡**
Diabetes	3.4 (1.4, 5.3)*	2.2 (-0.9, 5.3)	4.2 (1.2, 7.3)*	4.3 (0.1, 8.5)*
Random glucose (per 10 mg/dl)	0.2 (0.1, 0.4)**	0.1 (0.0, 0.3)	0.3 (0.1 ,0.5)*	0.4 (0.0, 0.7)*
HbA1c (%)	1.1 (0.5, 1.7)**	0.7 (-0.1,1.5)	1.4 (0.5, 2.3)*	1.6 (-0.1, 3.3)

n = number of eyes*,* *β* = beta coefficient, 95% CI = 95% confidence interval, HbA1c = glycosylated haemoglobin

†Diabetes, random glucose and HbA1c were assessed separately, in respective multivariable model adjusted for age, gender, corneal curvature, axial length, body mass index, ethnicity (only for overall group), and duration of diabetes (duration was denoted as ’0’ for participants without diabetes).

‡*β* for diabetes represents adjusted difference in CCT between persons with and without diabetes (reference group); *β* for random glucose and HbA1c models represent adjusted change in CCT per unit change in random glucose and HbA1c, respectively.

*Denotes $P < 0.05$.

**Denotes $P < 0.001$.

© 2019 Luo X-Y et al. *JAMA Network Open.*

eFigure 1. Flowchart of information through different phases of the meta-analysis.

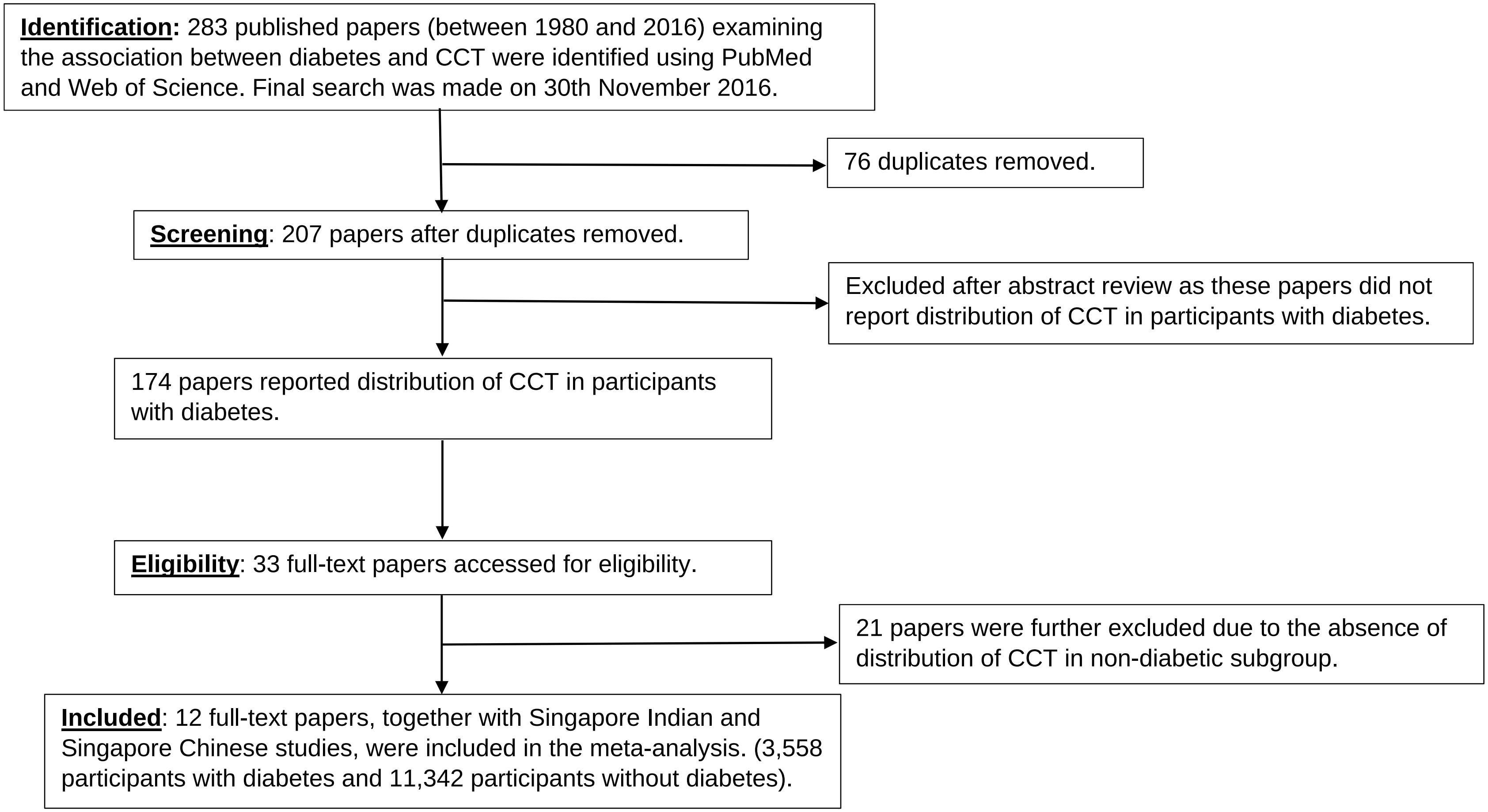

© 2019 Luo X-Y et al. *JAMA Network Open.*

eFigure 2. Flowchart of study subjects.

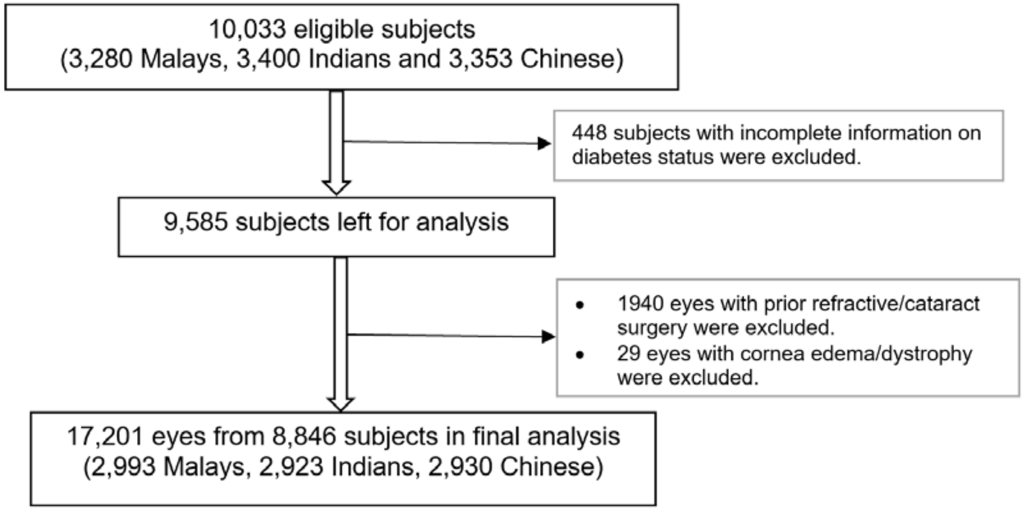

© 2019 Luo X-Y et al. *JAMA Network Open.*
